# Circadian control of sleep-related neuronal activity in lizards

**DOI:** 10.1093/pnasnexus/pgad481

**Published:** 2023-12-29

**Authors:** Sho T Yamaguchi, Sena Hatori, Koki T Kotake, Zhiwen Zhou, Kazuhiko Kume, Sam Reiter, Hiroaki Norimoto

**Affiliations:** Department of Cellular Pharmacology, Graduate School of Medicine, Hokkaido University, Sapporo 060-8638, Japan; Department of Cellular Pharmacology, Graduate School of Medicine, Hokkaido University, Sapporo 060-8638, Japan; Department of Cellular Pharmacology, Graduate School of Medicine, Hokkaido University, Sapporo 060-8638, Japan; Department of Cellular Pharmacology, Graduate School of Medicine, Hokkaido University, Sapporo 060-8638, Japan; Graduate School of Pharmaceutical Sciences, Nagoya City University, Nagoya 467-8603, Japan; Computational Neuroethology Unit, Okinawa Institute of Science and Technology (OIST) Graduate University, Okinawa 904-0495, Japan; Department of Cellular Pharmacology, Graduate School of Medicine, Hokkaido University, Sapporo 060-8638, Japan

**Keywords:** circadian rhythms, REMS/SWS alternation, *Pogona vitticeps*

## Abstract

Although diurnal animals displaying monophasic sleep patterns exhibit periodic cycles of alternating slow-wave sleep (SWS) and rapid eye movement sleep (REMS), the regulatory mechanisms underlying these regular sleep cycles remain unclear. Here, we report that in the Australian dragon *Pogona vitticeps* exposed to constant darkness (DD), sleep behavior and sleep-related neuronal activity emerged over a 24-h cycle. However, the regularity of the REMS/SWS alternation was disrupted under these conditions. Notably, when the lizards were then exposed to 12 h of light after DD, the regularity of the sleep stages was restored. These results suggest that sleep-related neuronal activity in lizards is regulated by circadian rhythms and that the regularity of REMS and SWS cycling is influenced by daytime light exposure.

**Significance Statement**

The influence of circadian rhythms on the regular REMS/SWS alternation in sleep remains unclear. By recording sleep-related neuronal activity in the diurnal lizard *Pogona vitticeps* under constant darkness (DD) conditions, we revealed the presence of circadian regulation of the sleep/wake cycle. Furthermore, we found that REMS/SWS alternation was disrupted under DD conditions and restored by 12 h of light exposure, thus demonstrating circadian regulation and sensory modulation of sleep patterns in *P. vitticeps* and offering valuable insights into the regulatory mechanisms of REMS/SWS alternation.

## Introduction

Diurnal animals with monophasic sleep patterns exhibit species-specific cycles of slow-wave sleep (SWS) alternating with rapid eye movement sleep (REMS) (1–6). Humans typically have inter-REMS intervals averaging approximately 90 min, with four to six cycles occurring throughout an 8-h sleep (1). In contrast, marmosets have shorter sleep-stage cycles lasting approximately 50 min, with a range of 10 to 17 cycles during the night (2). A recent study revealed that in the diurnal reptile *Pogona vitticeps*, SWS and REMS regularly alternate with approximately 2-min cycles throughout the night, generating more than 300 SWS/REMS cycles (3). Although the cycle varies significantly across species, common neural mechanisms may be involved. However, the precise mechanisms responsible for maintaining these regular cycles remain elusive.

Sleep-wake patterns are predominantly regulated by the circadian clock, which maintains an ∼24-h sleep/wake rhythm even in the absence of external cues (7–9). Long-term activity recordings under constant dark (DD) conditions are commonly used to study circadian behavior. While these methods allow for the assessment of free-running rhythms in spontaneous activity, accurately tracking sleep-wake stages such as SWS, REMS, and wakefulness poses significant challenges. Several studies addressing this issue report that each vigilance stage, notably wakefulness and REMS, relies on circadian rhythms (10, 11). Nevertheless, to date, few studies have explored circadian effects on the dynamics of REMS/SWS alternation in diurnal animals. In this study, we focused on the impact of DD conditions on the regular alternation of REMS and SWS in *P. vitticeps*.

## Results

### Behavioral and pigmentation rhythms under DD conditions

**Fig. 1.**
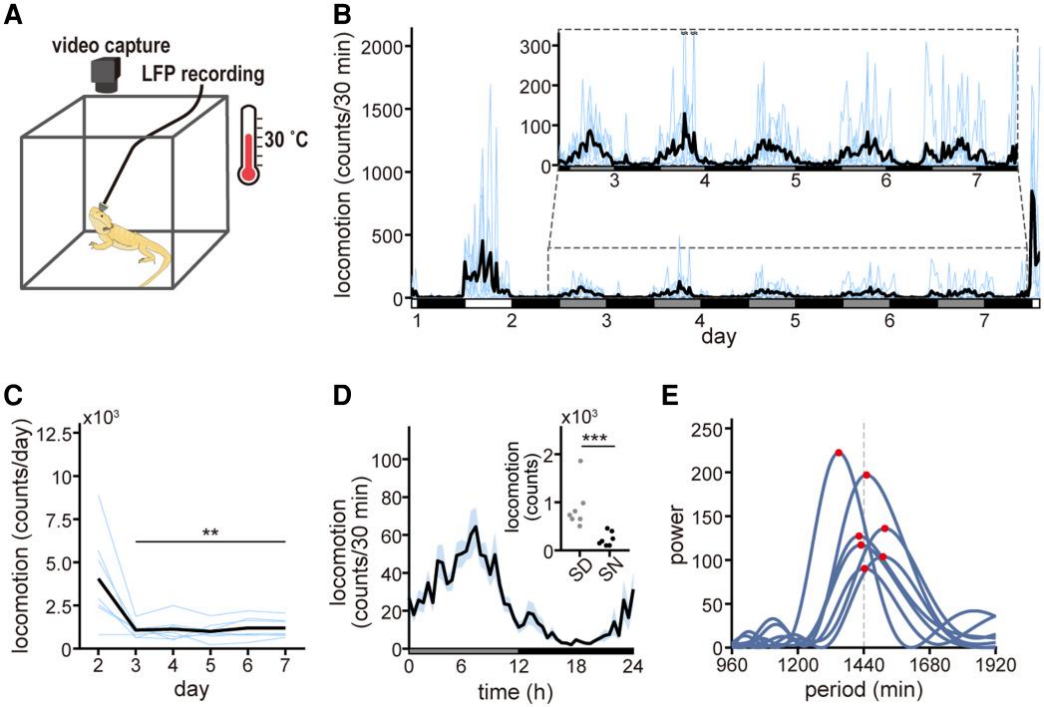
Behavioral rhythmicity under the DD condition. A) Experimental image. The lizards were maintained under a 12:12-h LD cycle, followed by constant DD for 5 days, followed by LD for 1 day. The ambient temperature was maintained at 30 °C. B) Shown is the 30-min interval locomotor activity during days 1–7. Blue lines are individual data; black lines are the average over all individuals (*n* = 7 animals). An enlarged view of the section enclosed by the gray dashed line is shown in the center. C) Total daily locomotor activity for each day. Results of one-way ANOVA followed by the Tukey–Kramer posthoc test vs. day 2 are as follows: day 3, *P* = 7.87 × 10^−4^, *Q _7, 7_* = 6.43; day 4, *P* = 0.001, *Q _7, 7_* = 6.31; day 5, *P* = 5.62 × 10^−4^, *Q _7, 7_* = 6.59; day 6, *P* = 0.001, *Q _7, 7_* = 6.16; day 7, *P* = 0.001, *Q _7, 7_* = 6.18. D) Activity profile under constant DD conditions. The 30-min interval locomotor activity of each individual under DD conditions is binned into 24-h intervals, and the average locomotion at each phase (horizontal axis) is calculated from all data (*n* = 35 recordings from seven animals for 5 days). The black line is the mean; blue shading is the SEM. The mean total activity in the SD and SN for each animal is shown in the top right (*n* = 7 animals). The activity during SD and SN were summed for each day and the mean of the total activity was calculated for each animal. ***P < 1.0 × 10^−7^ two-sided, by a two-sample hypothesis test using bootstrapping. E) Lomb–Scargle periodograms of locomotor activity under DD conditions for 5 days. The red circle on each periodogram indicates the peak of the estimated power. The period lengths of the peaks are 1,349, 1,450, 1,517, 1,423, 1,430, 1,510, and 1,443 min, reading from top to bottom.

In the DD experiments, the lizards were exposed to 12:12-h light-dark (LD) conditions (days 1 and 2), followed by 5 days of constant darkness (DD), followed by the LD condition for 1 day (Figs. 1A and S1). Lizards in the control group were maintained under LD conditions for 6–8 consecutive days (referred to as “LD experiments,” Fig. S1). The temperature was maintained at 30 °C. Videos and local field potentials (LFP) were recorded continuously during the experiments. Food and water were withheld during the experiments. The effect of food and water deprivation for 8 days on body weight was remarkably subtle, and these differences were not significant between the LD and DD experiments (Fig. S1, −3.14 ± 0.50% for the LD group and −3.56 ± 0.53% for the DD group, mean ± SEM).

To quantify the locomotor activity of the lizards, we used a frame subtraction method on video data to detect body movements. These movements were converted into locomotor counts (Fig. S2; see the Material and methods section). Under LD conditions, the lizards exhibited a diurnal behavioral pattern, being most active during the light phase and displaying minimal movement during the dark phase (Fig. S3). Despite the lack of food and water, total daily locomotor activity remained constant throughout the LD experiments (Fig. S3). Under DD conditions, the lizards showed reduced locomotor activity compared with LD conditions (Fig. 1B). Total daily locomotion during the DD condition (days 3–7) was significantly lower than that during the LD condition (day 2) (Fig. 1C). Nevertheless, lizard behavior under DD conditions exhibited a diurnal rhythm, with a significant difference in total activity between the inferred subjective day (SD) and subjective night (SN, Fig. 1D). Repeated-measures analysis of variance (RM-ANOVA) and the Lomb–Scargle periodogram demonstrated the presence of a behavioral rhythm under DD conditions with a ∼24-h cycle in all individuals (Fig. 1E). The brightness of the body surface also exhibited circadian fluctuation in four of seven lizards under DD conditions (Fig. S4), consistent with previous research that showed that about 50% of *P. vitticeps* individuals exhibited a circadian rhythm in body brightness (12). These results indicate that lizard behavior is regulated by endogenous circadian rhythms.

### The circadian sleep/wake cycle under the DD condition

**Fig. 2.**
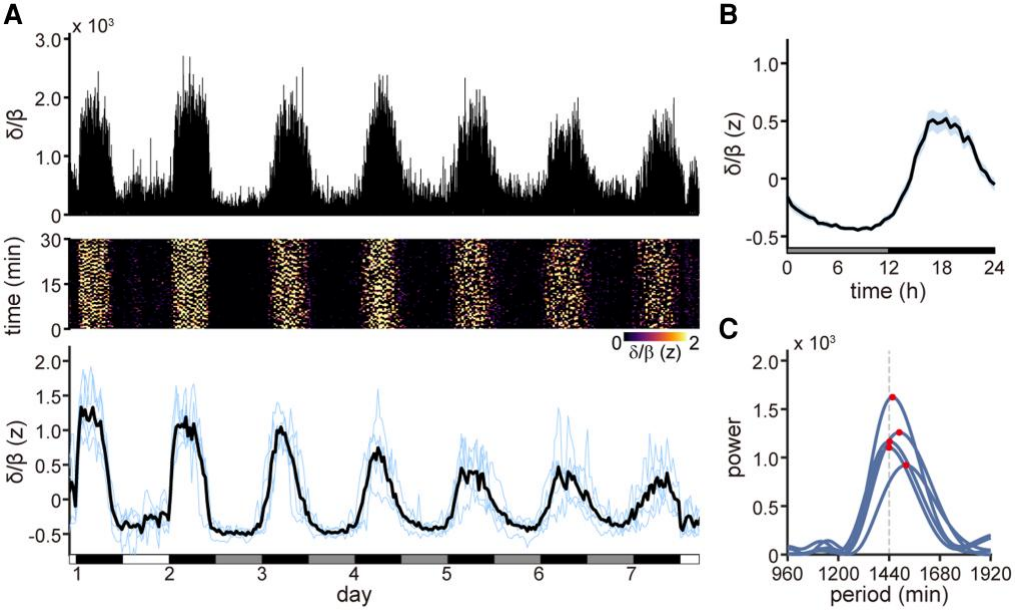
Circadian rhythmicity in the power of the LFP under the DD condition. A) Top: a representative trace of the δ/β ratio during days 1–7. Middle: a representative image showing the normalized δ/β ratio during days 1–7. Each vertical column represents a 30-min segment, progressing from bottom to top, and successive 30-min segments run continuously from left to right. Bin size, 1 s. Bottom: the mean δ/β ratio at 30-min intervals during days 1–7. The data from each lizard was aligned and then standardized using *z*-scores. Blue lines represent individual data; black line represents the average of all individuals (*n* = 5 animals). White, gray, and black horizontal bars indicate light, SD, and dark (or SN) phases, respectively. B) The profile of the z-scored δ/β ratio under DD conditions. The 30-min interval mean δ/β ratios (z-scored) of each individual under DD conditions are binned into 24-h intervals, and the mean δ/β ratio at each phase (horizontal axis) is calculated from all data (*n* = 25 sessions of five recordings for 5 days from five animals). Blue shading, SEM. C) Lomb–Scargle periodogram of the δ/β ratio under DD conditions for 5 days. The red circles indicate the peaks of the estimated power. The period lengths of the peaks are 1,456, 1,488, 1,442, 1,440, and 1,520 min, reading from top to bottom.

To examine the effects of DD conditions on neuronal activity during sleep, we recorded LFPs from the claustrum or the adjacent dorsal ventricular ridge (DVR), the dominant noncortical pallial domain of sauropsid brains (13–16), because sleep/wake stages in lizards are distinguished by neuronal activity in these regions (3, 16, 17). We used the ratio of delta (0.1–3 Hz) power to beta (10–30 Hz) power, referred to as the delta/beta (δ/β) ratio, as the basis of the classification of sleep stages (3). Under DD conditions, δ/β oscillated on a 24-h cycle, with the maximum at SN (Fig. 2A and B). RM-ANOVA and Lomb–Scargle periodogram analyses confirmed the circadian rhythmicity of the δ/β ratio (Fig. 2C). These findings indicate the presence of a circadian rhythm in the electrophysiological sleep-wake cycle of lizards.

### The regularity of the REMS/SWS alternation is diminished under DD conditions

**Fig. 3.**
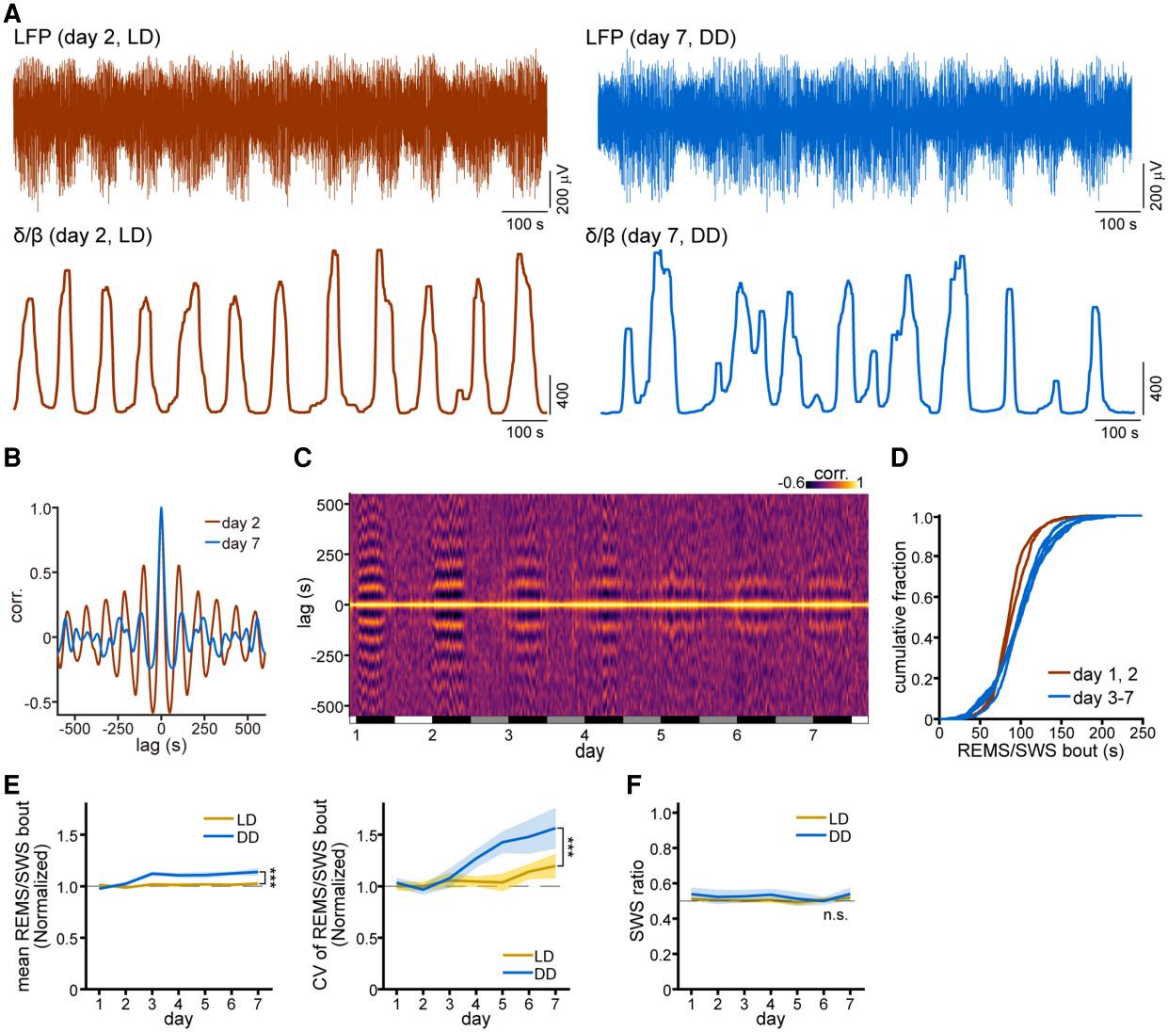
Modification of REMS/SWS alternations under DD conditions. A) Top: short segments of LFP (top) and δ/β ratio (bottom), displaying the REMS/SWS alternations (low/high δ/β ratio), on days 2 (left), and 7 (right). The δ/β ratio data are shown after median filtering (20-s window). B) Auto-correlogram of the δ/β ratio shown in (A). C) Representative heatmap for the auto-correlogram of the filtered δ/β ratio at 30-min intervals. Each vertical column represents the auto-correlogram of the δ/β ratio for 30 min from the time point indicated on the horizontal axis. The auto-correlograms of successive 30-min segments of the δ/β ratio run continuously from left to right. The white, gray, and black horizontal bars indicate light, SD, and dark (or SN) phases, respectively. D) Cumulative fractions of REMS/SWS bout length for each day from day 1 to day 7. Data from each individual are aggregated by day, and the cumulative fraction is calculated (*n* = 5 animals for each day). E) Mean (left) and CV (right) of REMS/SWS bout lengths for each day (*n* = 5–7 recordings each from four animals for LD experiments, and *n* = 5 recordings each from five animals for DD experiments). The data from each individual are normalized by division by the mean of the corresponding data recorded on days 1 and 2. The results of the two-way ANOVA, LD vs. DD, are as follows: *F*_1, 68_ = 36.35; ***P < 1.0 × 10^−7^ for the mean REMS/SWS bout (left); *F*_1, 68_ = 13.35; ***P = 5.05 × 10^−4^ for the CV of the REMS/SWS bout lengths (right). Blue shading, SEM. F) SWS ratio during sleep (*n* = 5–7 recordings each from four animals for LD experiments, and *n* = 5 recordings each from five animals for DD experiments). n.s., not significant by two-way ANOVA, LD vs. DD; *F*_1, 68_ = 2.70, *P* = 0.105. Blue shading, SEM.

Next, we examined the regularity of the REMS and SWS alternations under different conditions. Under the LD condition, on day 2, the δ/β ratio oscillated regularly with a fixed period (Fig. 3A). However, under the DD condition, the oscillation in δ/β became irregular and arrhythmic by day 7. The auto-correlogram of the δ/β ratio revealed that under DD conditions, the patterns were irregular and their amplitudes were dampened in comparison with those recorded under LD conditions (Fig. 3B). The sliding auto-correlogram revealed that the δ/β oscillation occurred predominantly during the night, irrespective of LD or DD conditions (Fig. 3C), but the peaks of the auto-correlogram were less distinct under DD conditions. These data suggest that REMS/SWS alternations became unstable under DD conditions. This inference is further supported by the cumulative distribution of REMS/SWS bouts, the interval between a given SWS bout and the subsequent SWS onset. Under the DD condition, the REMS/SWS bouts were shifted to longer durations, and the slopes of the cumulative plots were moderate compared with those observed in the LD condition (Fig. 3D), indicating that the REMS/SWS bouts are variable and relatively long under the DD condition. Notably, we did not observe any prolongation or instability of the REMS/SWS under the LD condition (Fig. 3E), indicating that the changes were specifically associated with the DD condition. Despite the changes in sleep architecture, the proportions of SWS and REMS did not differ between the LD and DD conditions (Fig. 3F), suggesting that the long-term visual deprivation and/or decreased locomotion associated with the DD condition disrupts the regularity of the REMS/SWS cycles.

### Restoration of the REMS/SWS alternations after 12-h light exposure

**Fig. 4.**
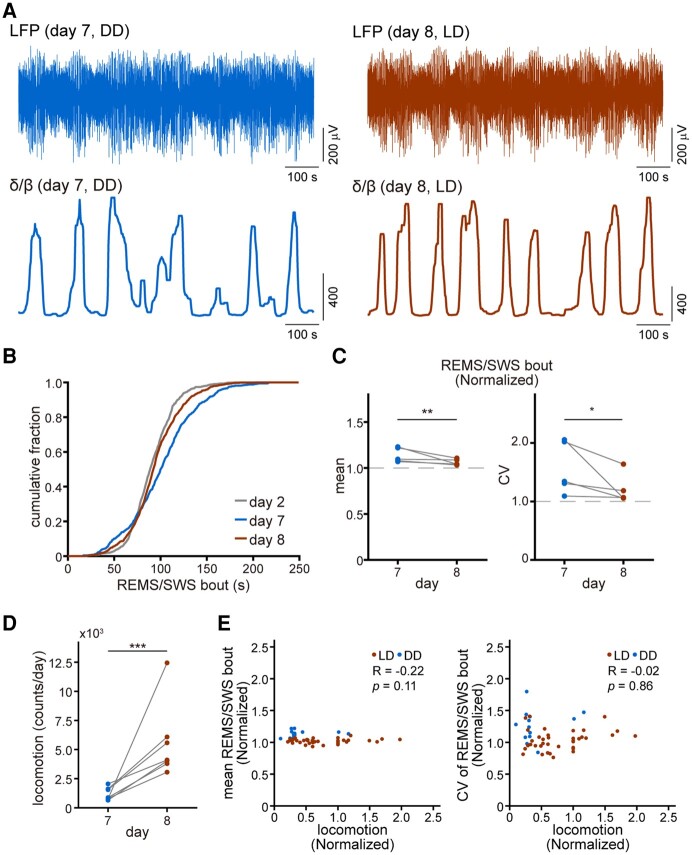
Restoration of the REMS/SWS alternation upon return to LD conditions. A) Short segments of LFP (top) and δ/β ratio (bottom), displaying the alternations in REMS/SWS (low/high δ/β ratio), on day 7 (left) and 8 (right). B) Cumulative fraction of REMS/SWS bout length for days 2, 7, and 8. Data from each individual are aggregated by day, and the cumulative fraction is calculated. C) Mean (left) and CV (right) of REMS/SWS bout lengths for days 7 and 8 (*n* = 5 animals). The data from each individual are normalized by division by the mean of the corresponding data recorded between days 1 and 2. **P = 0.008; *P = 0.036, by a two-sample hypothesis test using bootstrapping (one-sided). D) Total daily locomotion on days 7 and 8 (*n* = 7). ***P < 1.00 × 10^−7^ by a two-sample hypothesis test using bootstrapping (one-sided). E) The mean (left) and CV (right) of REMS/SWS bout length are plotted against the amount of daily locomotion (*n* = 52 sessions, comprising six recordings of LD experiments from four animals and three recordings of DD experiments from three animals). Red dots represent data from days with the LD condition (days 2 and 8 of DD experiments and from day 2 to day 8 of LD experiments); blue dots represent data from days with the DD condition (from days 3 to 7 of DD experiments). *R*, Pearson's correlation coefficient; *P*, confidence level to reject the null hypothesis.

Finally, we examined whether the lengthened and unstable REMS/SWS alternations observed under DD conditions were restored after returning to LD conditions, during which time the lizards were more active and provided with visual information. The LFP on day 8 showed more regular REMS/SWS alternations compared with those of the DD condition on day 7 (Fig. 4A). The cumulative distribution of REMS/SWS bouts on day 8 resembled that on day 2 (LD condition) rather than that on day 7 (DD condition) (Fig. 4B). The increased mean and coefficient of variation (CV) of the REMS/SWS bouts observed under the DD condition decreased significantly on day 8 (Fig. 4C). Locomotor activity also significantly increased on day 8 (Fig. 4D). These results indicate that a 12-h light exposure restores the longer and more irregular REMS/SWS alternations that emerge under the DD condition.

We also examined whether locomotion influenced REMS/SWS alternation by computing the correlation coefficients between the total daily locomotion and the properties of the REMS/SWS bouts. Neither the mean nor the CV of the REMS/SWS bouts was correlated with locomotion (Fig. 4E). These results suggest that changes in REMS/SWS alternations under the DD condition were primarily driven by visual deprivation rather than by a reduction in locomotor activity.

## Discussion

We here describe the circadian and ultradian sleep-related rhythms in lizards under DD conditions. Under DD conditions, *P. vitticeps* exhibits circadian behavioral rhythms. Furthermore, SWS and REMS occurred during the SN, even in the absence of external cues (e.g. light, temperature, and feeding). These findings indicate that lizard locomotion and sleep/wake cycles are regulated by an endogenous circadian rhythm.

Intriguingly, under the DD condition, the REMS/SWS alternations became longer and more variable than under the LD condition. The disrupted rhythms were restored when the lizards were returned to the LD condition. The decreased locomotor activity observed under DD conditions was also reinstated by exposure to light; however, the amount of locomotor activity did not correlate with the duration or variation of the REMS/SWS alternations. These results suggest that the REMS/SWS cycle observed at night may be maintained by light exposure during the day, which is consistent with previous studies showing disrupted sleep-stage transitions in sensory-deprived ponies, which is known to be diurnal (18). In addition, several studies using nocturnal or crepuscular animals have investigated the effects of DD conditions on each sleep stage. These studies imply that the effect of DD conditions on the REMS/SWS alternation may be widely observed across species (19–22, but see Ref. 23). However, it is important to note that the animals used in these studies are nocturnal or crepuscular and their sleep is largely fragmented, with frequent occurrences of wakefulness, which may also contribute to the modifications of each sleep stage.

A possible mechanism for changes in sleep architecture caused by visual deprivation is the impact of visual deprivation on the circadian clock system (24). In squirrel monkeys, rhythmic REMS/SWS alternations with a period of 60 min have been observed. However, damage to the suprachiasmatic nucleus, a dominant circadian pacemaker in the mammalian brain, disrupted the REMS/SWS switching process (6). In *P. vitticeps*, decreased light input to the homologous master clock may have led to irregular REMS/SWS alternations. Further experiments, such as lesioning the master clock in lizards (25–29) or simultaneously recording in lizards the LFPs in the DVR and the neuronal activity in the master clock, would allow a deeper understanding of how the circadian clock system influences the regulation of REMS/SWS alternation.

In conclusion, we demonstrated that the sleep of *P. vitticeps* is regulated by the internal circadian clock but that the clock alone, without the presence of light, is not sufficient to maintain the regularity of the REMS/SWS alternation. Our findings reveal additional similarities in sleep between lizards and other amniotes, emphasizing the utility of lizards in sleep research. Further investigations using reptiles could enable us to access in detail the fundamental mechanisms of REMS/SWS alternation that are conserved across species.

### Limitations of the study

We only examined *P. vitticeps* in our study; however, it is necessary to investigate additional animal species to determine whether our findings can be generally applied to other animals.

## Materials and methods

### Animals

Ten lizards (*Pogona vitticeps*; seven males, two females, and one unknown), weighing 150–300 g, were obtained from external breeders and housed individually in reptile terraria (45 × 45 × 30 cm) maintained on a 12; 12-h light/dark cycle. The lights-on and lights-off times were referred to as 0 and 12 h, respectively. A halogen spotlight for basking was placed above the terraria and turned on between 3 and 9 h each day. The lizards had access to water ad libitum and were fed either vegetables (bok choy, carrots, and lettuce) or crickets on alternate days. Calcium and vitamin D3 supplementation (GEX Corporation, Japan) were provided in the vegetables.

All procedures involving animals were approved by the Animal Care and Use Committee of Hokkaido University (21-0092).

### Surgery

The day before surgery, the lizards were administered meloxicam (Boehringer Ingelheim Animal Health Japan; 0.2 mg/kg s.c.). On the day of the surgery, the animal was initially placed in a box filled with isoflurane (Mylan Inc., USA) and, slightly later, topical anesthesia (xylocaine; Sandoz Pharma, Japan) was administered around the trachea. After induction of deep anesthesia, the animal was intubated via the trachea and placed in a stereotactic apparatus. During surgery, anesthesia was maintained with isoflurane (0.5–3 vol%), and body temperature was maintained at around 32 °C using a heating pad. The heart rate was monitored using an ultrasonic blood flow sensor.

A craniotomy was performed around the parietal eye, and the overlying skin was disinfected using a 7.5% povidone–iodine solution (Meiji, Japan) before removal with a scalpel. The dural and arachnoid layers covering the forebrain were removed using fine forceps, and the pia was gently removed over the area targeted for electrode insertion. The exposed skull was covered with ultraviolet (UV)-curing glue, and the bare ends of two insulated stainless-steel wires (0.005 in, A-M Systems, USA) were placed subdurally to serve as the electrical reference and ground, respectively, and fixed with UV-curing glue.

To insert the electrodes, a stainless-steel wire (A-M Systems) was mounted on an OpenEphys shuttleDrive (30) or a 3D-printed custom microdrive based on the shuttleDrive and secured to a stereotactic adaptor. The electrodes were lowered about 1–1.5 mm from the brain surface, targeting the DVR. The brain surface was covered with Dura-gel (Neurotech, UK). After connecting the reference to the ground, the skull, craniotomy, and microdrive were tightly secured using UV-curing glue and dental cement. After surgery, the lizards were injected s.c. with meloxicam (0.2 mg/kg), released from the stereotactic apparatus, and maintained on a heating pad until fully recovered from anesthesia.

### Recording

Before recording, the lizards were habituated to an acrylic cage (30 × 30 × 30 cm) and placed in an incubator for at least one night. On the initial day of recording, the lizard was fed crickets in addition to normal feeding. One to two hours before lights went off, the lizard was moved into an acrylic cage inside an incubator, in which the ambient temperature was maintained at 30 °C, and the recording for day 1 was then started. For DD experiments, the lizards were exposed to 12:12-h LD conditions (day 2), followed by 5 days of DD conditions (days 3–7), then returned to LD conditions for 1 day (day 8). For the LD experiments, the lizards were maintained under the LD condition for 6–8 consecutive days, including day 1. The lizards in both groups were not provided with food or water during the experiments.

The body weight of the lizards was measured before and after the experiment. To calculate the changes in body weight, the body weights after the experiments were subtracted from those at baseline (before or a week after the experiments), followed by division by the body weights at baseline, and expressed as percentages (%). Body weight data from LD experiments lasting 6 or 7 days were excluded from the analysis.

Video recordings were conducted using a Universal Serial Bus camera (BFS-U3-16S2M-CS; Teledyne FLIR, USA) positioned above the acrylic cage, and illumination was provided by an infrared transmitting plate. The video data were acquired using SpinView software (Teledyne FLIR, Wilsonville, Oregon, USA) at a rate of 10 frames per second.

The LFP recordings were performed using the OpenEphys system and 32-channel head stages (Intan Technologies, Los Angeles, CA, USA). The recordings were referenced to one of the wires. Signals were sampled at 1 kHz and band-pass filtered at 0.1–100 Hz.

### Video analysis

To detect locomotion, frames were converted to grayscale, and each of the two consecutive frames was subtracted. The resulting subtraction images were binarized, and connected components with fewer than 10 pixels in each frame were excluded. The number of pixels that exhibited changes between two successive frames (referred to as the pseudo-velocity) was then calculated. The pseudo-velocity was thresholded to remove lights-on/off events. The difference between the pseudo-velocities at two consecutive time points was computed as the pseudo-acceleration. Locomotor activities were counted as events when the pseudo-acceleration exceeded 2 × standard deviations.

For the extraction of body brightness, the videos were downsampled to 1 Hz. The body parts of the lizards were tracked using DeepLabCut (31). Before extracting the pixel intensity, the videos were converted to grayscale. The sum of the pixel intensities obtained from a specific area on the back of the lizard was normalized to the intensity obtained from the background. After detrending, data points that deviated by more than 3 × local standard deviation from the local mean over a 30-min interval or data points with a pseudo-velocity exceeding 2 × standard deviation were excluded. The remaining data points were linearly interpolated following standardization to *z*-scores.

### LFP analysis and sleep staging

After binning the data in 10-s windows and 1-s steps, the power spectrum for each bin was calculated using the fast Fourier transform algorithm. The δ/β ratio was calculated by dividing the mean spectrum over frequencies lower than 3 Hz by the mean spectrum over frequencies between 10 and 30 Hz in each bin. The median filtered (20-s window) δ/β ratio was used for the calculation of auto-correlograms. Electrostatic noise was removed by setting a visually guided threshold on the LFP plot.

Sleep-stage analysis was performed using the data obtained during a 4-h period centered around the peak of the median filtered (6-h window) δ/β ratio. The onsets/offsets of the SWS/REMS alternations were calculated by upward/downward threshold crossing of the median filtered (20-s window) δ/β ratio. The threshold was set as the average between the 100 smallest and 100 largest points of the median filtered (1,000-s window) δ/β ratio. The interval between two consecutive SWS onsets was computed as the duration of a REMS/SWS alternation. REMS/SWS bouts that deviated by more or less than 2 × standard deviations from the mean were excluded from further analysis. The SWS ratio was computed as the intervals between the SWS onsets and the subsequent REMS onsets summed over 4 h, and then dividing by 4 h.

### Circadian rhythm analysis

The data were summed (for locomotion) or averaged (for body color and δ/β ratio) over 30-min intervals, then binned into 24-h intervals. The phase profiles of each parameter were calculated by averaging all the data at each time point. RM-ANOVA was performed using individual profile data to estimate whether the individual was rhythmic (*P* < 0.05) or not (32). In addition, the data were smoothed by calculating the 1-min interval sum (for locomotion) or average (for body color and δ/β ratio). The Lomb–Scargle periodogram was constructed using 1-min interval data, and a peak in the periodogram ranging from 1,200 to 1,680 min was detected. A peak in the Lomb–Scargle periodogram was defined as significant if it had a power greater than that of the minimum of the peaks from individuals estimated as rhythmic by RM-ANOVA. Any individual parameter exhibiting a significant peak in the Lomb–Scargle was classified as “rhythmic.” The data were z-scored before the calculation of profiles for body color, the δ/β ratio, and the Lomb–Scargle periodograms for body color.

### Calculation of correlation coefficients

The mean and CV of the REMS/SWS bout durations from each individual were divided by the mean of the corresponding data recorded between days 1 and 2. The locomotor activity of each individual was scaled by the corresponding data recorded on day 2. Pearson's correlation coefficient was calculated after outliers had been deleted, and outliers were detected using the Grubbs’ test.

### Statistical analysis

The presented data are the mean ± SEM or individual data with/without means. Data were analyzed using MATLAB version 9.12.0 (R2022a; The MathWorks Inc., Natick, MA, USA) (33) and Python (*Python 3 Reference Manual,* 2009; CreateSpace; Scotts Valley, CA, USA) (34), as described above and in the figure legends.

## Supplementary Material

pgad481_Supplementary_Data

## Data Availability

The data are openly available in Mendeley Data at https://data.mendeley.com/datasets/v7kkwgnyn5/1.
