# Supplementary material for: Circadian control of sleep-related neuronal activity in lizards
Source: PNAS Nexus. 2023 Dec 29;3(1):pgad481. doi: 10.1093/pnasnexus/pgad481 (PMC10783807; doi:10.1093/pnasnexus/pgad481)
Supplement: pgad481_Supplementary_Data [file pgad481_supplementary_data.pdf]

Supporting Information for

Circadian Control of Sleep-Related Neuronal Activity in Lizards.

Sho T. Yamaguchi, Sena Hatori, Koki T. Kotake, Zhiwen Zhou, Kazuhiko Kume,

Sam Reiter, Hiroaki Norimoto

Correspondence to: Hiroaki Norimoto, PhD and Sho Yamaguchi, PhD

Email: norimoto@pop.med.hokudai.ac.jp and sho.yamaguchi723@gmail.com

This PDF file includes:

Figures S1 to S4

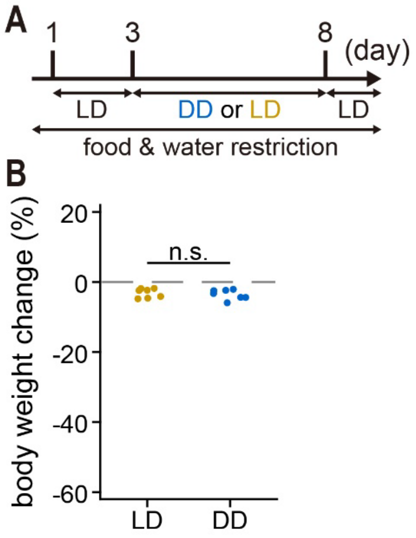

Fig. S1. Experimental protocol and body weight changes. (A) Schematic diagram of the experimental protocol. Lizards in the DD group were maintained on a 12:12-h light-dark cycle (LD), followed by constant darkness (DD) for 5 days, followed by LD for 1 day. The LD group was maintained under LD conditions for 6–8 successive days. During these experiments, the lizards were kept under restricted food and water intake. (B) Body weight changes after food and water restriction for 8 days under LD or DD conditions. LD experiments, n = 7 recordings each from 2 animals; DD experiments, n = 7 recordings each from 7 animals; n.s., not significant by two-sample hypothesis test using bootstrapping (two-sided): p = 0.540.

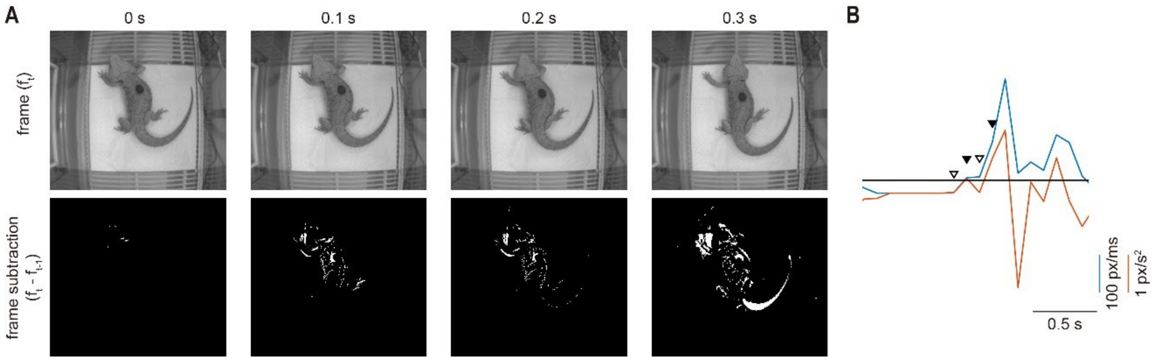

Fig. S2. Methodology for behavioral monitoring. (A) Examples of original frames (top) and frame subtraction results (bottom). The frame subtraction images were binarized and filtered to remove salt-and-pepper noise. (B) The graph displays the pseudo-velocity (blue) and pseudo-acceleration (orange). Black line, threshold (2 × standard deviation) for activity detection based on numbers of changed pixels; inverted triangles, time points of frames shown in (A); black inverted triangles, time points when locomotor activity is detected.

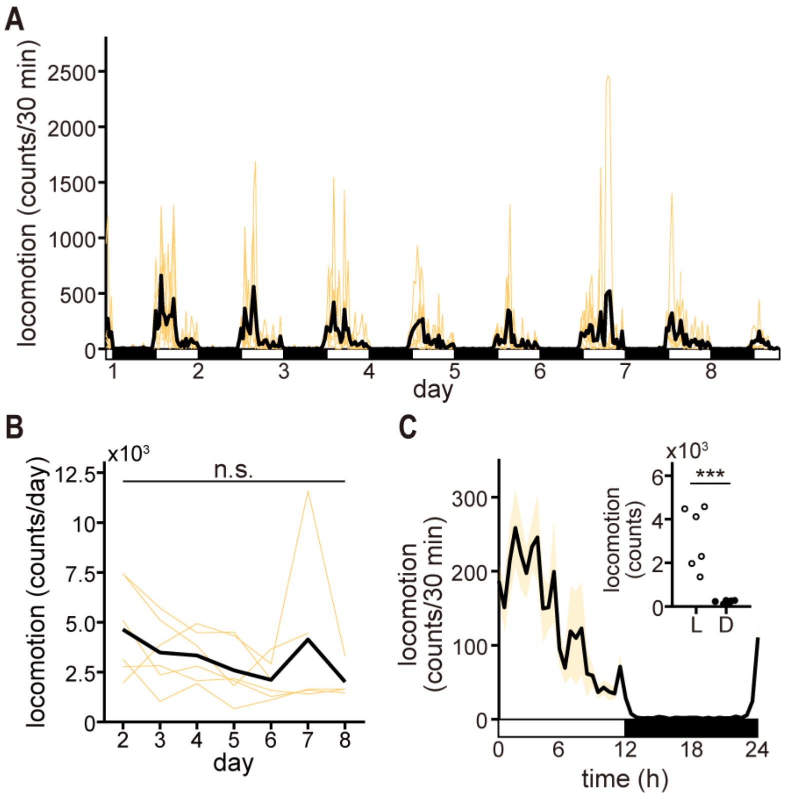

Fig. S3. Locomotor activity of lizards under LD conditions. (A) The 30-min-interval locomotor activity, all measurements. Yellow lines: individual data; black line: the average across individuals (n = 4–6 recordings from 3 animals). The white and black horizontal bars represent tne light and dark phases, respectively. (B) Total daily locomotor activity for each day. n.s.; not significant by one-way ANOVA: $F_{6, 88}$ = 1.18, p = 0.344. (C) Activity profile under LD conditions. The 30-min-interval locomotor activity of each individual under LD conditions was binned into 24-h epochs, and the average locomotion at each phase (horizontal axis) was calculated from all data (n = 39 sessions of 6 recordings for 7 days from 3 animals). Black line, mean; yellow shade, SEM. White

and black horizontal bars, light and dark phases, respectively. The mean total activity in the light (L) and dark (D) phases for each animal is shown in the top right (n = 6 recordings from 3 animals). The activity during L and D phases were summed for each day and the mean of the total activity were calculated for each recording. ***, $p < 1.00 \times 10^{-7}$ by a two-sample hypothesis test using bootstrapping (two-sided).

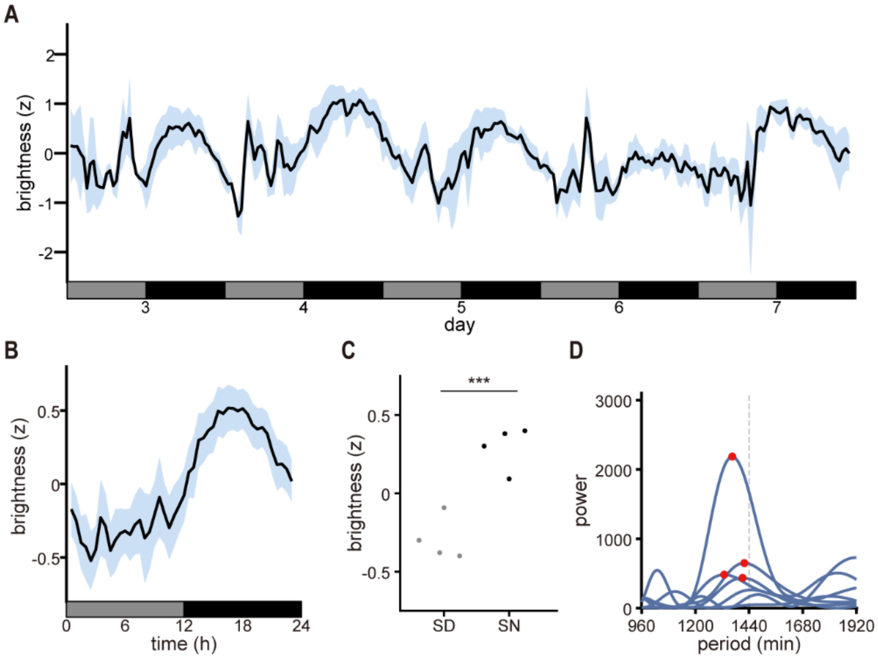

Fig. S4. Changes in body brightness under DD conditions. (A) The 30-min-interval mean brightness of the body surface (z-scored), all measurements. Black line, an average of rhythmic individuals (n = 4 animals); blue shade, SEM. Gray and black horizontal bars represent subjective day and subjective night phases, respectively. (B) The profile of body surface brightness under constant darkness (DD) conditions. The 30-min-interval z-scored mean brightness of the body surface from each rhythmic individual under DD conditions was binned into 24-h epochs, and the average brightness against phase (horizontal axis) was calculated from all data (n = 20 sessions of 4 recordings for 5 days from 4 animals). Gray and black horizontal bars represent the subjective day and subjective night phases, respectively. (C) Mean Z-scored body surface brightness in the subjective day (SD) and subjective night (SN) for each animal (n = 4 animals). The z-scored

brightness during SD and SN were averaged for each day, and the mean for each animal was then calculated. ***$p < 1.00 \times 10^{-7}$ by a two-sample hypothesis test using bootstrapping (two-sided). (D) Lomb–Scargle periodogram of body surface brightness under the DD condition for 5 days. The red circle on each curve indicates the significant peak of estimated power. The period lengths of the peaks are 1365, 1419, 1329, and 1411 min from top to bottom. The three remaining peaks were not significant.
